# Translational Regulation of Duplicated Gene Expression Evolution in Allopolyploid Cotton

**DOI:** 10.3390/genes15091130

**Published:** 2024-08-27

**Authors:** Guiling Fu, Haotian Luo, Juqing Jia, Mingming Hou, Guanjing Hu

**Affiliations:** 1 College of Agriculture, Shanxi Agricultural University, Taigu 030801, China; 2 Shenzhen Branch, Guangdong Laboratory of Lingnan Modern Agriculture, Key Laboratory of Synthetic Biology, Ministry of Agriculture and Rural Affairs, Agricultural Genomics Institute at Shenzhen, Chinese Academy of Agricultural Sciences, Shenzhen 518120, China; 3 National Key Laboratory of Cotton Bio-Breeding and Integrated Utilization, Institute of Cotton Research, Chinese Academy of Agricultural Sciences, Anyang 455000, China; 4 Western Research Institute, Chinese Academy of Agricultural Sciences, Changji 831100, China

**Keywords:** polyploidization, translatome, transcriptome, cotton, Ribo-seq, translation efficiency

## Abstract

Polyploidy, a prevalent event in plant evolution, drives phenotypic diversification and speciation. While transcriptional changes and regulation in polyploids have been extensively studied, the translational level impact remains largely unexplored. To address this gap, we conducted a comparative transcriptomic and translatomic analysis of cotton leaves from allopolyploid species *G. hirsutum* (AD_1_) and *G. barbadense* (AD_2_) relative to their model A-genome and D-genome diploid progenitors. Our data revealed that while allopolyploidization significantly affects the transcriptional landscape, its impact on translation was relatively modest, evidenced by a narrower expression range and fewer expression changes in ribosome-protected fragments than in mRNA levels. Allopolyploid-specific changes commonly identified in both AD_1_ and AD_2_ were observed in 7393 genes at either transcriptional or translational levels. Interestingly, the majority of translational changes exhibited concordant down-regulation in both ribosome-protected fragments and mRNA, particularly associated with terpenoid synthesis and metabolism (352 genes). Regarding translational efficiency (TE), at least one-fifth of cotton genes exhibit translational level regulation, with a general trend of more down-regulation (13.9–15.1%) than up-regulation (7.3–11.2%) of TE. The magnitude of translational regulation was slightly reduced in allopolyploids compared with diploids, and allopolyploidy tends to have a more profound impact on genes and functional associations with ultra-low TE. Moreover, we demonstrated a reduced extent of homeolog expression biases during translation compared with transcription. Our study provides insights into the regulatory consequences of allopolyploidy post-transcription, contributing to a comprehensive understanding of regulatory mechanisms of duplicated gene expression evolution.

## 1. Introduction

Polyploidy, the whole genome duplication (WGD) event, has been a fundamental and recurrent force in angiosperm evolutionary history, profoundly influencing genome structure, function, and organismal complexity [1,2]. By doubling or multiplying genome content, polyploidy provides a reservoir for genetic innovation, leading to phenotypic diversification and adaptation [3,4]. A key finding of the last decade is the profound impact of polyploidy on transcriptomic landscapes [5,6,7]. Studies have reported substantial transcriptomic rewiring in polyploid species, including biased homoeolog expression, conditional gene regulation, and transgressive expression levels. These transcriptional changes often underlie phenotypic and adaptive variations upon polyploidy formation. However, despite extensive research on transcriptome and proteome [8], the consequences of polyploidy at the translational level remain largely unexplored.

Ribosome profiling (Ribo-seq) is a high-throughput technique that maps ribosome positions on mRNA, offering a snapshot of active translation by sequencing mono-ribosome-protected mRNA fragments or ribosome footprints [9]. By analyzing the distribution and abundance of these footprints, researchers can quantitatively assess translation rates, identify translation start sites, and uncover the full repertoire of translated open reading frames (ORFs) [10]. Beyond its utility in identifying translated regions, Ribo-seq enables the study of translational regulation, including the impact of *cis*-regulatory elemental, ribosome pausing, protein, and environmental cues on protein synthesis [11,12,13]. Moreover, by comparing Ribo-seq data with transcriptomic data, translational efficiency (TE), a metric that reflects the coupling of transcription and translation could be measured [14,15].

The application of Ribo-seq has rapidly expanded across various plant systems. In *Arabidopsis*, for example, translatome and RNA sequencing revealed tightly regulated translation that poorly correlated with transcriptional changes during immune induction, uncovering novel immune regulators [16]. Similarly, translational reprogramming demonstrated a crucial layer of stress responses in crop species, including rice (*Oryza sativa*) [17,18], potato (*Solanum tuberosum*) [14], citrus (*Citrus reticulata*) [19], and maize (*Zea mays*) [15]. Beyond its role in stress responses, Ribo-seq has been instrumental in investigating allele-specific translation in hybrids, revealing the intricate interplay between genetic variation and translational regulation [13,20]. These studies highlighted the potential role of translational regulation in shaping heterosis, giving hybrid offspring superior traits compared to their parents. Although progress has been made in understanding the impact of polyploidy on translational regulation, such as in soybean (*Glycine max*) [21], the role of translational control in gene expression evolution during allopolyploidization remains largely unexplored.

Cotton, a globally significant fiber crop, represents a model system for studying allopolyploidization [22]. The allopolyploid cotton genome was formed approximately 1–2 million years ago through hybridization and subsequent genome doubling of A- and D-genome diploid progenitors. Previous research on cotton gene expression evolution accompanying allopolyploidization has primarily focused on transcriptomics [23,24] and proteomics [25,26], with limited insights from translational regulation. To address this gap and elucidate the impact of allopolyploidization on translational regulation, we conducted a comprehensive analysis of gene expression at both the transcriptional and translational levels in allopolyploid cotton *G. hirsutum* (AD_1_) and the representatives of its progenitor diploid species *G. arboreum* (A_2_) and *G. raimondii* (D_5_).We also included public data from another allopolyploid species *G. barbadense* (AD_2_) [27] to expand our comparative analysis. Our study aimed to investigate the extent and mode of translational regulation in response to allopolyploidization, how it differs from transcriptional regulation, and its role in shaping homoeolog expression patterns between subgenomes in allopolyploid cotton.

## 2. Materials and Methods

### 2.1. Plant Materials

Three *Gossypium* species were used in the present study: one allopolyploid species *G. hirsutum* cv. TM-1 (AD_1_) and two diploid species representing the model diploid progenitors, *G. arboreum* cv. Zhongya 1 (A_2_) and *G. raimondii* PI 530899 (D_5_). Seeds were germinated in a growth chamber in darkness under controlled conditions (28 ± 1 °C and 60 ± 5% humidity). After germination, seedlings were cultivated with a 16 h light/8 h dark photoperiod cycle under the same controlled condition for six weeks. Leaf tissue from the fourth to fifth true leaves was collected from three biological replicates (5 plants per replicate) of each genotype, immediately frozen in liquid nitrogen, and stored at −80 °C.

### 2.2. Ribosome Profiling (Ribo-seq)

Ribosome-protected fragments (RPFs) were isolated using a modified protocol [28]. Briefly, 1 g of leaf tissue was ground in liquid nitrogen with 10% [*w/w*] polyvinylpolypyrrolidone poly (vinylpolypyrrolidone) in liquid nitrogen and resuspended in to 5 ml of lysis buffer (150 mM Tris-HCl pH 8.0, 40 mM KCl, 20 mM MgCl_2_, 2% [*v/v*] polyoxyethylene, 0.4% [*w/v*] sodium deoxycholate, 1.5 mM dithiothreitol, 50 μg/mL chloramphenicol, 50 μg/mL cycloheximide, and 2% [*v/v*] polyvinylpyrrolidone). Adding 25 μL DNase I to a final concentration of 10 units/mL, the mixture was incubated on ice for 30 min. After centrifugation at 4 °C for 10 min at 8000× *g*, the supernatant was transferred to a new centrifuge tube and further centrifuged at 4 °C for 10 min at 16,000× *g*. The supernatant was layered onto an 8 mL cushion (150 mM Tris-HCl pH 8.0, 40 mM KCl, 20 mM MgCl_2_, 5 mM EGTA, 1.5 mM dithiothreitol, 50 μg/mL chloramphenicol, 50 μg/mL cycloheximide, and 1.75 M sucrose) and subjected to ultracentrifugation at 4 °C, 257,000× *g* for 3 h in a ultracentrifuge (Beckman Coulter, Optima XE-100, USA) using a Type 70 Ti Rotor. The pellet was washed twice with pre-cooled ddH2O and resuspended in 300 μL RNase I digestion buffer (10 mM Tris-HCl pH 8.0, 100 mM NaCl, 10 mM EDTA, and 5 mM EGTA,1% [*w/v*] SDS). The resuspended mixture was transferred to a new tube and centrifuged at 1000× *g* at 4 °C for 30 s. Six units of RNase I were added to the supernatant and incubated at room temperature for 1.5 h. For rRNA depletion from RPFs, a Ribo-pool rRNA Depletion Kit (siTOOLs, p-k024-000031, DER) was used. The obtained RNA fragments were recovered using the RNA Clean & Concentrator-5 kit (Zymo, R1014, USA) and purified using the 15% denatured PAGE gel (7 M Urea, 0.5 × TBE, 15% [*v/v*] Acrylamide-Bisacrylamide, 0.08% [*v/v*] Ammonium persulfate substitute, and 0.05% [*v/v*] TEMED) targeting 27–34 nt in size, according to the protocol [29].

To construct Ribo-seq libraries from purified and size-selected RPFs, the phosphate group of RPFs was treated with T4 PNK, followed by 3′ adaptor (5′-rAppGATCGGAAGAGCACACGTCT-NH2) ligation using truncated T4 RNA ligase 2 (NEB). The RNA strand with added adapters was denatured at 65 °C along with the designed RNA primers. Subsequently, reverse transcription was carried out using SuperScript II (ThermoFisherScientific, Waltham, MA, USA) and an RT primer (5′-GATCGTCGGACTGTAGAACTCTGAACGTGTAGATCTCGGTGGTCGCCGTATCATT/iSp18/CACTCA/iSp18/CAGACGTGTGCTCTTCCGATCT) at 42 °C for 1.5 h. RPFs were circularized with Circligase ssDNA ligase (Epicentre, CL4111K, USA) and amplified by PCR. Finally, PCR amplification to generate a 150 bp cDNA library was performed using E × Taq (14 cycles, 60 °C annealing, and primer sequences are listed in Table S1), which was sequenced using Illumina NovaSeq 6000 by Pasenno Biotechnology Co. (Shanghai, China).

### 2.3. RNA Sequencing (RNA-Seq)

Total RNA was extracted using the TIANGEN Total RNA extraction kit (TIANGEN X0817). RNA-seq libraries were prepared using the Illumina TruSeq Stranded mRNA-seq library preparation kit (refer to online protocol accessed on 26 March 2023) for paired-end sequencing (150 bp × 2) on an Illumina HiSeq 2500 platform (Novogene Technology Co., Beijing, China).

### 2.4. Ribo-seq and RNA-seq Data Processing

In addition to data generated from A_2_, D_5_, and AD_1_, public cotton leaf RNA-seq and Ribo-seq data for another allopolyploid species, *G. barbadense* (AD_2_), were downloaded from the National Center for Biotechnology Information (NCBI) SRA depository (details in Supplemental Table S2) to enable comparisons across different polyploid genotypes. Public cotton ribosomal RNA (rRNA) sequences were retrieved from NCBI and subjected to de-redundancy using CD-HIT (v4.8.1) [30] to generate a reference set for rRNA filtering.

Quality control, adapter trimming, and low-quality read filtering were performed using FASTP (0.23.4) [31]. Bowtie2 (v2.3.5.1) [32] was used to remove rRNA reads, utilizing the non-redundant cotton rRNA sequence set as a reference. To facilitate direct comparison between allopolyploid and diploid datasets, the number of reads in the matched cleaned data from A_2_ and D_5_ diploids was adjusted to equal amounts and then combined to generate a “Combined Diploid” dataset (A_2_D_5_). The processed data from AD_1_, AD_2_, and A_2_D_5_ were then subjected to further analysis. 

Processed reads from RNA-seq were aligned to the *G. hirsutum* cv. TM-1 UTX (v2.0) reference genome [33]. Similarly, using Hisat2 (v2.2.1) [34] with default parameters (details provided), read counts and transcripts per million (TPM) values were quantified for each gene using the featureCounts function from the Rsubread package (v2.16.1) [35].

Processed reads from Ribo-seq were aligned to the same reference genome using STAR (v2.7.9a) [36] with parameters ‘—outFilterMismatchNmax 1—outFilterMultimapNmax 1’ to retain only uniquely mapped reads with at most one mismatch. The R function *featureCounts* was used to obtain read counts and TPM values. BAM files from STAR alignment were subjected to determine read length distribution and three-nucleotide (3 nt) periodicity using RiboCode (v1.2.15) [37].

### 2.5. Analysis of Gene Expression at Transcriptional and Translational Levels

Differential gene expression analysis was performed using the DESeq2 package in R (v1.44.0) [38] to identify genes with significant expression changes between allopolyploids (AD_1_ and AD_2_) and the combined diploid dataset (A_2_D_5_). Genes with adjusted *p*-values < 0.05 by Benjamini–Hochberg correction [39] were considered differentially expressed. Pairwise comparisons were conducted between AD_1_ vs. A_2_D_5_ and AD_2_ vs. A_2_D_5_ at both transcriptional and translational levels. To investigate the coordinated regulation of gene expression, genes were categorized into eight groups by cross-tabulating their differential expression patterns (i.e., increased, decreased, and unchanged relative to A_2_D_5_) between two levels. Except for the category with no change in expression at both levels, functional enrichment analysis of Gene Ontology (GO) terms was performed for genes in each of the other eight groups using R-clusterProfiler (v4.8.3) [40], with *p*-values adjusted for multiple testing using the Benjamini–Hochberg method. A threshold of an adjusted *p*-value < 0.05 was applied to identify significantly enriched GO terms. Functional protein annotation performed by Revigo was employed to summarize and visualize significant GO terms (v 3.4.4) [41].

### 2.6. Calculation of Translational Efficiency

TE was calculated for each gene as the log2 ratio of RPF to mRNA expression levels: TE = log2 (RPF/mRNA). To account for potential library size differences, both RPF and mRNA expression data were normalized using the median of ratios normalization method built-in DESeq2. Pairwise comparisons between matched Ribo-seq and RNA-seq samples for each genotype were conducted using DESeq2 to obtain log2FoldChange values as TE estimates. Genes with an adjusted *p*-value < 0.05 were considered to have significantly different TE. A positive TE value indicates higher translation efficiency relative to transcription, while a negative value suggests lower translation efficiency. Genes exhibiting extreme TE changes (|TE| > 6) were subjected to Gene Ontology (GO) enrichment analysis using R-clusterProfiler (v4.8.3) [39]. The R package pheatmap (v1.0.12) was utilized for visualization [42].

### 2.7. Analysis of Homoeolog Expression Bias (HEB)

HEB was defined as the unequal expression of homoeologous gene copies in the allopolyploid [5]. In allopolyploid cotton (AD genome), the direction of bias was determined by the subgenome (At or Dt) exhibiting higher expression. The At and Dt homoeologous relationships within the allopolyploid *G. hirsutum* reference genome were previously inferred [43]. 

To assess HEB at the transcriptional and translational levels, expression levels (mRNA and RPF) were compared between At and Dt homoeologs using DESeq2. Genes with adjusted *p*-values < 0.05 (Benjamini–Hochberg correction) were considered to exhibit significant HEB. The magnitude of HEB was quantified as the log2fold-change in expression between homoeologs. At the genomic scale, the prevalence of bias towards a specific subgenome was evaluated to assess genome-wide HEB.

To assess HEB at the translational level, TE values were compared between At and Dt homoeologs using Student’s *t*-test. Significant differences in TE between homoeologs were determined using Fisher’s combined *p*-value < 0.05.

## 3. Results

### 3.1. Transcriptional and Translational Profiling of Diploid and Allopolyploid Cotton

Ribo-seq data were generated from seedling leaves of *G. arboreum* (A_2_), *G. raimondii* (D_5_), and *G. hirsutum* (AD_1_) with an average of 61.1 million, 43.5 million, and 75.4 million reads per replicate, respectively (Table S2). After removing low-quality reads and rRNA, an average of 18.1 million clean reads remained per sample, representing 30.8% of the total. The average unique mapping rate ranged from 11.2% to 39.6%, tetraploid (AD_1_) having the lowest rate, most likely due to its higher level of sequence homology compared with diploids A_2_ and D_5_. To facilitate direct comparisons between parental diploids and allopolyploids, we combined the clean reads of A2 and D5 into a ‘Combined Diploid’ dataset (A_2_D_5_) and used it for subsequent analyses, which exhibited 14.5–16.5% unique mapping rates, comparable to those of AD_1_.

**Figure 1:**
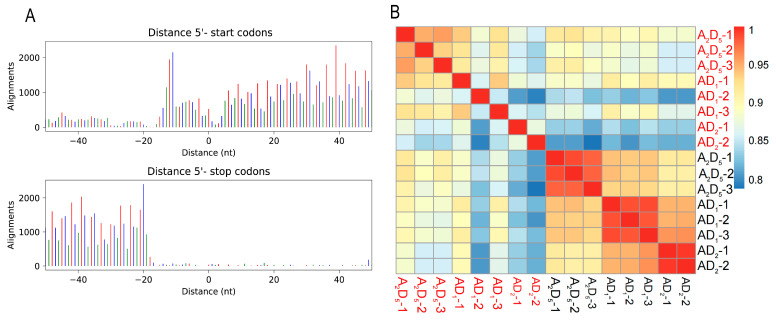
Characteristics of ribosome profiling data in diploid and allopolyploid cotton. (**A**) Three-nucleostide (3-nt) periodicity flanking the annotated translation start and stop sites in *G. raimondii* (D_5_) cotton genome. Red, blue, and green bars represent reads mapped to the first (expected), second, and third reading frames, respectively. (**B**) Pearson correlation coefficients matrix of Ribo-seq (red) and RNA-seq (black) data from diploids combined (A_2_D_5_) and two allopolyploid species *G. hirsutum* (AD_1_) and *G. Barbadense* (AD_2_); the latter was obtained from a previously published study [27].

To validate Ribo-seq data quality, we examined RPF (Ribosome Protected Fragment) sizes, which ranged from 26 to 32 nucleotides (Figure S1), consistent with known cotton Ribo-seq data [27]. The RPFs exhibited a periodicity of 3 nt (Figure 1A and Figure S2), a typical characteristic of ribosome profiling [43]. Pairwise Pearson correlation coefficients were calculated between all samples to examine data reproducibility between replicated experiments, including a previously published dataset from *G. barbadense* (AD_2_) leaves (Figure 1B) [27]. PCA clustering analysis of the samples showed good clustering among the experimental data, except for one AD_1_ sample (AD_1_-2), which had poor correlation (Figure S3). Therefore, AD_1_-2 was excluded from subsequent analyses.

For RNA-seq, on average, A_2_, D_5_, and AD_1_ cotton leaf samples yielded 43.5 million, 47.7 million, and 51.7 million raw reads, respectively (Table S3). The clean read pairs obtained per sample ranged from 39 to 57 million, with an average unique mapping read rate of 87.4% (Table S3). Diploid data from A_2_ and D_5_ were combined into the A_2_D_5_ dataset as for the Ribo-seq data. Pearson correlation coefficients ranging from 0.79 to 0.99 were observed between transcriptome and translatome data (Figure 1B). The results above indicated that our experiments have yielded sufficiently reliable data.

Notably, expression variances were higher in transcriptomes than in translatomes (Figure S4; variance of log10 (TPM): 0.914 vs. 0.686), potentially indicating a wider expression range in mRNA levels than translated proteins.

### 3.2. Concordantly Decreased Transcription and Translation Accompanying Allopolyploidization

**Figure 2:**
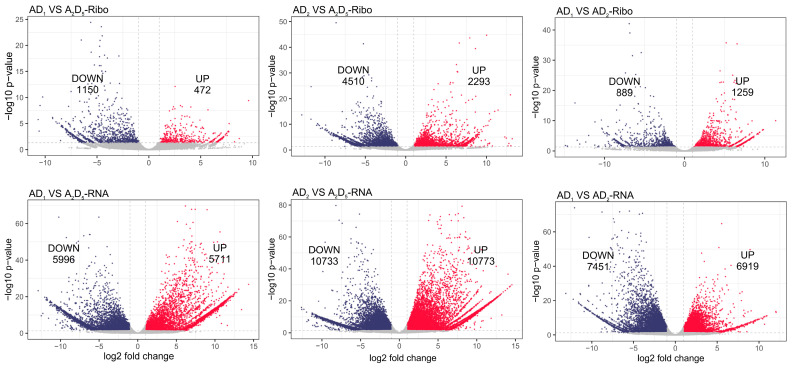
Differential expression analysis between AD_1_, AD_2_ and A_2_D_5_.

**Figure 3:**
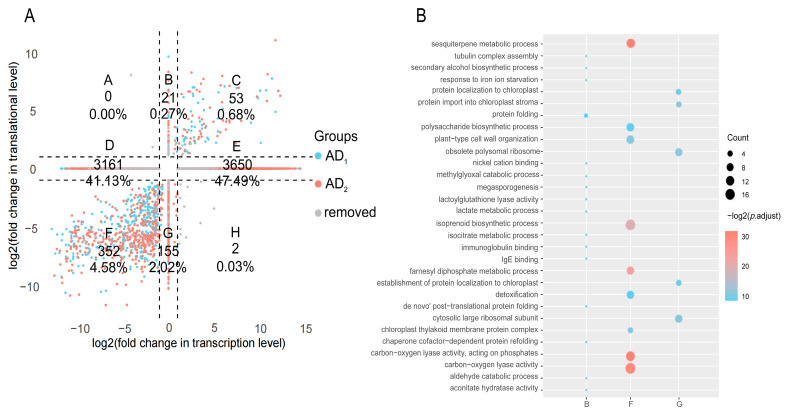
Expression patterns of AD_1_ and AD_2_ relative to A_2_D_5_. (**A**) Classification of allopolyploid-specific changes commonly identified in AD_1_ and AD_2_, by cross-tabulating three expression patterns (increased, decreased, and no changes) between RNA-seq and Ribo-seq. For example, category C of 53 genes exhibits increased transcription and translation in both AD_1_ and AD_2_ relative to A_2_D_5_. (**B**) Gene Ontology analysis for the genes exhibiting allopolyploidy changes at the translational levels. Significant GO terms were identified for categories B, F, and G.

To study gene expression changes during allopolyploidization, we conducted differential gene expression analyses between diploid and tetraploid cotton using RNA-seq and Ribo-seq data (criterion: |log2fold change| ≥ 1 and FDR < 0.05). We identified significant differences as follows: comparing AD_1_ to A_2_D_5_, 11,707 (15.6%, 5711 up-regulated and 5996 down-regulated) transcriptional changes and 1622 (2.1%, 472 up-regulated and 1150 down-regulated) translational changes were identified (Figure 2). Comparing AD_2_ to A_2_D_5_, more DEGs were identified at both levels, with 21,506 (28.7%, 10,773 up-regulated and 10,733 down-regulated) transcriptional changes and 6803 (9.1%, 2293 genes up-regulated and 4510 down-regulated) translational changes. Between AD_1_ and AD_2_, 14,370 (19.2%, 6919 up-regulated and 7451 down-regulated) transcriptional changes and 2148 (2.9%, 1259 up-regulated and 889 down-regulated) translational changes were identified. Concordantly, fewer DEGs were identified at the translational level by Ribo-seq. Regarding the four inter-ploidy contrasts (i.e., AD_1_ vs. A_2_D_5_ and AD_2_ vs. A_2_D_5_ at either transcriptional or translational levels), we identified a total of 7686 genes exhibiting allopolyploidy changes common to both tetraploids AD1 and AD2 (Figure S5, numbers in red). Of these, 189 genes showed common changes only at the translational level, 7078 genes showed changes only at the transcriptional level, and 419 genes showed changes at both levels. Considering only those exhibiting conserved up- or down-regulated changes relative to A_2_D_5_, allopolyploid-specific regulation was inferred for 7393 genes, which were further categorized into eight groups (A-H) based on their expression changes in Ribo-seq and RNA-seq (each being increased, decreased, or no changes) (Figure 3A). 

We focus on the categories exhibiting allopolyploid-specific regulation at the translational level, including categories A, B, C, F, G, and H (582 genes, 7.9%). Among these, categories C (53, 0.7%) and F (352, 4.6%) exhibited concordant regulatory directions between transcription and translation in allopolyploids relative diploids, with a significant bias of decreased expression (more genes in category F than that in category C). To address biological functions involved in the translational regulation under allopolyploidization, we performed Gene Ontology (GO) enrichment analysis (Figure 3B). Category F was enriched terms associated with terpenoid synthesis and metabolism, indicating a negative impact of allopolyploidization on terpenoids at both transcriptional and translational levels. Category C was not enriched significant GO term.

Few transcripts were found exhibiting opposite regulatory directions between transcription and translation, as classified to category A (0 genes) or H (2 genes), nor enriched for GO terms. 

Categories B (21 genes, 0.3%) and G (155 genes, 2%) include genes with unchanged transcription levels but higher or lower translation in allopolyploids, respectively. Only category G was significantly enriched with GO terms related to ribosome function and protein import into chloroplasts. 

### 3.3. Impact of Allopolyploidy on Translational Efficiency

To investigate the impact of transcription on the translational change, we calculated the translation efficiency (TE) of each transcript to evaluate its utilization by ribosomes, which was defined as the ratio of translational to transcriptional expression levels [i.e., log2 (RPF/mRNA)]. A gene with TE = 0 is considered to have consistent transcriptional and translational products, while TE > 0 suggests the transcript underwent up-regulation at the translational level, and TE < 0 suggests translational down-regulation. 

**Figure 4:**
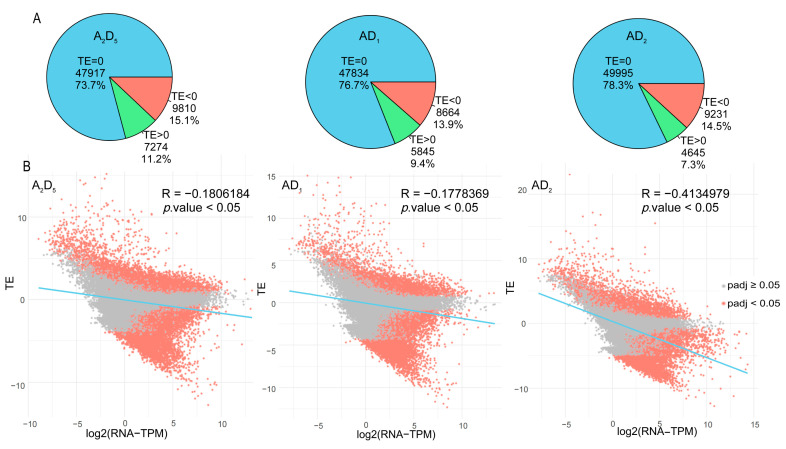
Comparing transcription efficiency (TE) between diploid and allopolyploid cottons. (**A**) Translational regulation in different genotype cotton. (**B**) Negative correlations between TE and mRNA levels.

We detected a total of 65,001 transcriptionally expressed genes (i.e., RNA-seq read counts above 1 in at least one biological replicate for the primary transcript of a given gene) in A_2_D_5_, with 9810 (15.1%) having TE < 0 and 7274 (11.2%) having TE > 0. In AD_1_, there were 62,343 genes, with 8664 (13.9%) TE < 0 and 5845 (9.4%) TE < 0. In AD_2,_ there were 63,871 genes, with 9231 (14.5%) and 4645 (7.3%) exhibiting TE < 0 and TE > 0, respectively (Figure 4A). These results indicate that at least one-fifth of genes exhibit translational level regulation, with a general trend of down-regulation rather than up-regulation of TE. Interestingly, the magnitude of translational level regulation was slightly reduced in allopolyploids compared with diploids.

To explore the relationship between TE and mRNA levels, we analyzed their correlations in each genotype. TE was significantly negatively correlated with transcriptional expression in A_2_D_5_, AD_1_, and AD_2_, with Pearson’s correlation coefficients (R) of −0.2, −0.2, and −0.4, respectively (Figure 4B). These negative values suggested the TE of transcripts and occupations of ribosomes are limited when mRNA levels are high, which was similar to the observations in maize and potato [14,15].

**Figure 5:**
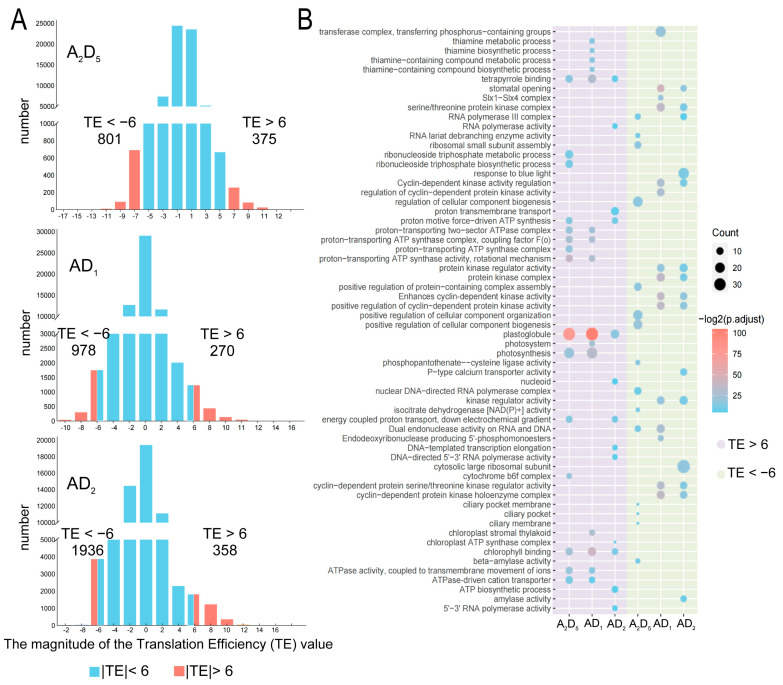
TE drastically changed transcripts between diploid and allopolyploid cottons. (**A**) Histogram distribution of TEs examined to choose the cutoff of |TE| > 6 for most significant translational regulated genes. (**B**) GO enrichment for transcripts with |TE| > 6.

We further analyzed the transcripts with the highest TE (>6) and the lowest TE (<−6) in A_2_D_5_ (1062 and 801 genes), AD_1_ (753 and 978) and AD_2_ (808 and 1936) (Figure 5A). GO analysis revealed that transcripts with TE > 6 in A_2_D_5_ were primarily enriched for energy metabolism-related terms, including ATPase activity, proton-transporting ATP synthase complex, ribonucleoside triphosphate metabolic process, and photosynthesis (Figure 5B). Similarly, these GO terms were enriched in TE drastically changed genes in AD_1_ and AD_2_, while additional RNA polymerase activity-related terms were enriched in AD_2_. This suggests that energy metabolism-related transcripts tend to be translated with high TE to increase protein production, either in diploid or in allopolyploid cotton. 

For transcripts with TE < −6, some terms of cellular component biogenesis and ciliary were predominantly enriched in A_2_D_5_ (Figure 5B). In contrast, both AD_1_ and AD_2_ were predominantly enriched for stomatal opening, cyclin-dependent protein, and GO terms related to serine/threonine protein kinase. This suggests that allopolyploidy has a profound impact on genes with low TE.

### 3.4. Reduced amount of Homoeolog Expression Biases at the Translational Level

To investigate the subgenomic contribution to gene expression, 22,889 ortho-homoeolog groups (OGs) were previously inferred between the At and Dt homoeologs in the AD_1_ reference genome. In the transcriptional level, we observed 23–37% of OGs exhibiting homeolog expression bias (HEB): A_2_D_5_ had 5285 (23%, 2574 A-bias vs. 2711 D-bias), AD_1_ had 5237 (22.9%, 2576 vs. 2661), and AD_2_ had 8407 (36.7%, 4165 vs. 4242), with no significant imbalance towards either the A- or D-subgenome. At the translational level, the number of OGs exhibiting HEB decreased to 3%-9%: A_2_D_5_ had 2224 (9.7%, 1115 vs. 1109), AD_1_ had 1013 (4.4%, 484 vs. 529), and AD_2_ had 562 (2.5%, 244 vs. 318), with only AD_2_ showing significantly more D-biases of translational expression (Table 1). 

When comparing TEs, there were 1076 (4.7%, 391 vs. 685), 625 (2.7%, 320 vs. 305), and 547 (2.4%, 237 vs. 310) significant differences between At and Dt in A_2_D_5_, AD_1_, and AD_2_, respectively. Both A_2_D_5_ and AD_2_ showed significant D-biased translational level regulation (Table 1). 

These results indicated that allopolyploidization may have reduced the subgenomic differences at the translational level, leading to fewer biases at this level. Additionally, a D-genome dominant translational regulation was observed in AD_2_, which may reflect species-specific translational regulation with respect to subgenomic contribution.

## 4. Discussion

While translational regulation has emerged as a key player in shaping plant phenotypes and evolutionary trajectories, few studies have examined the translatomic dynamics of allopolyploidization [21]. In this study, we conducted comprehensive transcriptomic and translatomic analyses on allotetraploid cotton species *G. hirsutum* (AD_1_) and *G. barbadense* (AD_2_) relative to their two diploid progenitors, *G. aboreum* (A_2_) and *G. raimondii* (D_5_), to investigate translational regulation associated with cotton polyploidization. 

Our results revealed that the number of genes expressed at the transcriptional level is significantly greater than that at the translational level, consistent with general findings in other ribosome profiling studies [44]. These differences may partly result from the technical distinctions between Ribo-seq and RNA-seq. Ribosome-protected fragments (RPFs) captured by Ribo-seq represent only the mRNA segments actively engaged in translation on ribosomes, thereby reflecting the biological state at a specific moment [45]. Additionally, technical limitations of Ribo-seq can restrict its capacity to profile as many genes as standard RNA-seq. For instance, the complexity of Ribo-seq library preparation is prone to biases and can reduce overall library yield. Moreover, shorter read lengths (~20 nt) in Ribo-seq limits the ability to distinguish between transcripts with similar sequences, potentially leading to misidentification or underestimation of gene expression. 

Focusing on the 7393 genes exhibiting expression changes in both allopolyploids relative to their diploid progenitors, we examined the concordance and discrepancies between mRNA and RPF levels (Figure 3). Consistent with the observation that more differential expression changes were identified by RNA-seq than Ribo-seq (Figure 2), most allopolyploid-specific changes were detected only at the mRNA level (categories D&E: 88.6%), which is substantially higher than changes detected solely at the RPF level (categories B&G: 2.3%). While technical limitations cannot be entirely excluded, this large discrepancy highlights the complexity and multifactorial nature of gene regulatory control. Using an integrative approach to isolate translational regulation by calculating translational efficiency (TE), we found that over one-fifth of transcriptionally expressed genes in allopolyploid cotton exhibited translational regulation (TE ≠ 1), similar to the 23.8% observed in soybean [21], further underscoring the significant role of translational regulation. 

Despite the differences in profiling capacities between sequencing techniques, one important observation is that higher mRNA levels are generally associated with lower translational efficiency (TE) (Figure 4). This inverse relationship between transcriptional and translational regulation may suggest a regulatory mechanism in allopolyploid cells that balances resource and energy allocation to maintain cellular homeostasis.

In terms of homoeolog expression bias (HEB) in allopolyploids, we observed a reduced extent of HEB at the translational level compared to the transcriptional level (Table 1). This reduced bias in translational regulation could indicate a compensatory mechanism that ensures balanced gene expression between the subgenomes, thereby contributing to the stabilization of allopolyploid genomes. While unbalanced HEB favoring either the A- or D-subgenomes, also termed “subgenome dominance” [46], has been reported in previous transcriptomic and proteomic studies of cotton, our findings revealed equivalent numbers of A- and D-biased expressions in AD_1_ across mRNA, RPF, and TE levels. However, in AD_2_, more D-biased genes were observed than A-biased genes at both the RPF and TE levels. Earlier studies at the protein level, particularly in cotton seeds and root tips, also detected a higher number of D-biased genes compared to A-biased genes [25,26]. Further research is needed to elucidate the molecular mechanisms driving these biases and their biological significance in allopolyploid adaptation and evolution.

Compared to the parental divergence between the A- and D-diploid genomes, we observed a reduction in A- versus D-genome expression bias in both allopolyploids across all regulatory levels (mRNA, RPF, and TE). This reduction in expression bias, also reported in a soybean study on polyploid translational regulation [21], suggests that translational regulation may help minimize disparities in gene expression between the merged allopolyploid genomes and their diploid progenitors. This adjustment could represent an adaptive response of the allopolyploid genome to reconcile expression incompatibilities between different genomes, re-establishing expression equilibrium to maintain cellular functionality and homeostasis [47]. Alternatively, it could reflect a buffering effect of the shared cellular environment following genome merger. These expression adjustments likely involve multiple mechanisms, including DNA methylation [48], histone modifications [49], and transcription factor activities [50,51]. Future integrative analyses incorporating both translational and transcriptional level regulations are needed to further explore these mechanisms and their contributions to expression regulation in allopolyploid genomes.

## 5. Conclusions

In this study, we investigated the translatomic dynamics by comparing transcriptomic and translational data between a combined diploid (A_2_D_5_) dataset and two allopolyploid datasets (AD_1_ and AD_2_). Our research revealed a narrower expression range and fewer expression changes at the translational level compared to the transcriptional level. Approximately one-fifth of the cotton transcriptome, with a predominant trend towards TE downregulation. We observed a reduced magnitude of translational regulation in allopolyploids compared to diploids, and a more pronounced impact of allopolyploidy on genes and functional associations with ultra-low TE. Furthermore, our study demonstrates a reduced extent of homeolog expression bias at the translational compared to the transcriptional level. These findings provide novel insights into the complex regulatory consequences of allopolyploidization on gene expression and translation efficiency in cotton, and highlight the importance of considering both transcriptional and translational changes to fully comprehend the functional implications of polyploidization in plants. 

## Figures and Tables

**Table 1:** Homoeolog expression bias (HEB) genes on transcriptional and translational levels.

		A_2_D_5_		AD_1_		AD_2_	
		Number	Proportion	Number	Proportion	Number	Proportion
RNA	Total	5285	23.09%	5237	22.88%	8407	36.73%
	A > D	2574	11.25%	2576	11.25%	4165	18.20%
	A < D	2711	11.84%	2661	11.63%	4242	18.53%
	*p*-value *	0.0595		0.2402		0.401	
Ribo	Total	2224	9.72%	1013	4.43%	562	2.46%
	A > D	1115	4.87%	484	2.11%	244	1.07%
	A < D	1109	4.85%	529	2.31%	318	1.39%
	*p*-value *	0.8988		0.1574		0.001799	
TE	Total	1076	4.70%	625	2.73%	547	2.39%
	A > D	391	1.71%	320	1.40%	237	1.04%
	A < D	685	2.99%	305	1.33%	310	1.35%
	*p*-value *	2.20 × 10^−16^		0.5485		0.001801	

* Chi-square test was performed to compare the numbers of A > D and A < D.

## Data Availability

A_2_, D_5_, and AD_1_ data are deposited under the NCBI repository, accession number PRJNA1143193. AD_2_ data are available on the public database GRAND (Gossypium Resource and Network Database) at http://grand.cricaas.com.cn (accessed on 10 November 2023) [27].

## Supplementary Materials

The following supporting information can be downloaded at: https://www.mdpi.com/article/10.3390/genes15091130/s1, Figure S1. Ribosome-protected fragment (RPF) length histogram. Figure S2. Three-nucleotide periodicity flanking gene coding regions of Ribo-seq data from *G. arboreum* (A_2_), *G. raimondii* (D_5_), and *G. hirsutum* (AD_1_). Figure S3. Principal component analysis (PCA) of normalized read counts in TPM. Figure S4. Comparing variances between Ribo-seq and RNA-seq data. Figure S5. Venn diagram intersection of four DEG lists between diploid and allopolyploid cotton: AD_1_ vs. A_2_D_5_ and AD_2_ vs. A_2_D_5_ at either transcriptional or translational levels. Table S1. Primers for the construction of Ribo-seq library. Table S2. Ribo-Seq data. Table S3. RNA-Seq data. Table S4. Expressed gene number on Ribo-seq. Table S5. Expressed gene number on RNA-seq.
