# Supplementary material for: Translational Regulation of Duplicated Gene Expression Evolution in Allopolyploid Cotton
Source: Genes (Basel). 2024 Aug 27;15(9):1130. doi: 10.3390/genes15091130 (PMC11431062; doi:10.3390/genes15091130)
Supplement: Supplementary file 1 [file genes-15-01130-s001.zip › genes-3159492-supplementary.pdf]

Translational regulation of duplicated gene expression evolution in allopolyploid cotton

Supplementary Materials:

Figure S1. Ribosome protected fragment (RPF) length histogram.

Figure S2. Three-nucleotide periodicity flanking gene coding regions, of Ribo-seq data from *G.arboreum* (A_2), *G.raimondii* (D_5), and *G.hirsutum* (AD_1).

Figure S3. Principal component analysis (PCA) of normalized read counts in TPM.

Figure S4. Comparing variances between Ribo-seq and RNA-seq data.

Figure S5. Venn diagram intersection of four DEG lists between diploid and allopolyploid cottons: AD_1 vs A_2D_5 and AD_2 vs A_2D_5 at either transcriptional or translational levels.

Table S1. Primers for the construction of Ribo-seq library

Table S2. Ribo-Seq data

Table S3. RNA-Seq data

Table S4. Expressed gene number on Ribo-seq

Table S5. Expressed gene number on RNA-seq

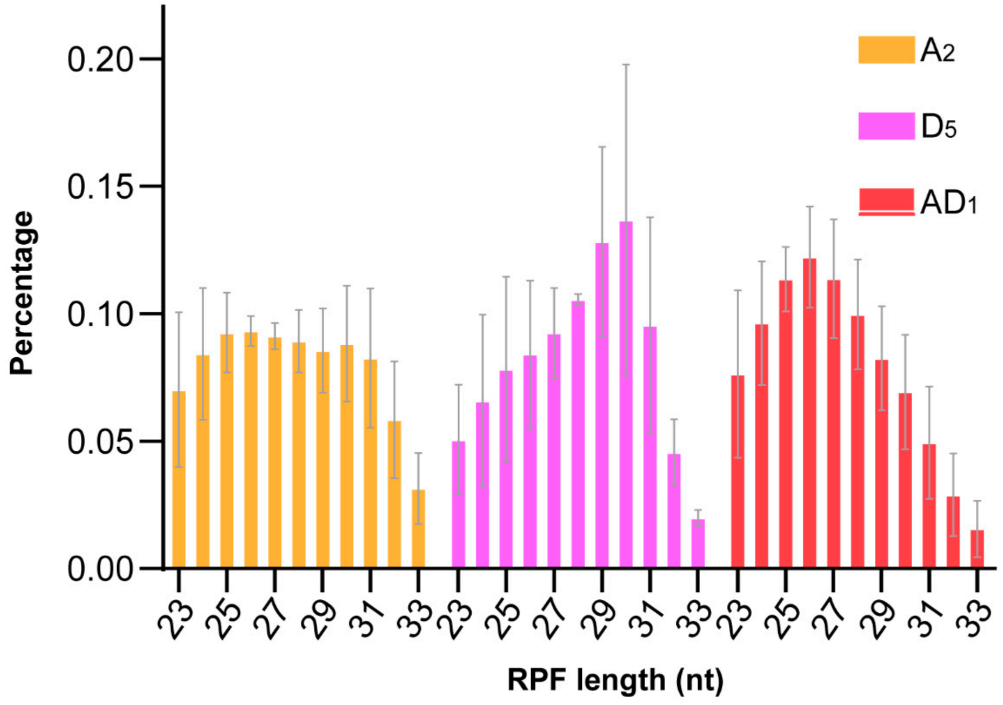

Figure S1. Ribosome protected fragment (RPF) length histogram.

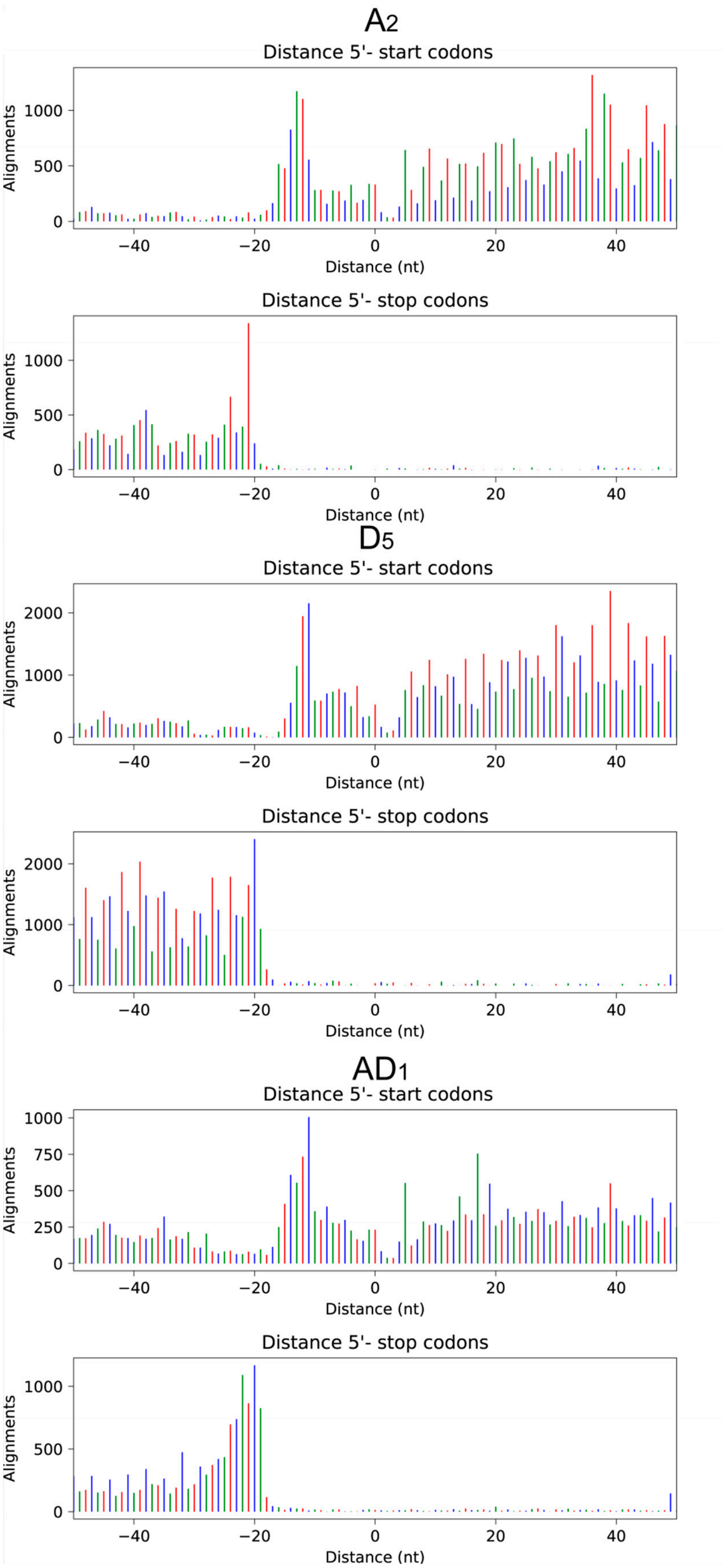

Figure S2. Three-nucleotide periodicity flanking gene coding regions, of Ribo-seq data from *G.arboreum* (A$_2$), *G.raimondii* (D$_5$), and *G.hirsutum* (AD$_1$).

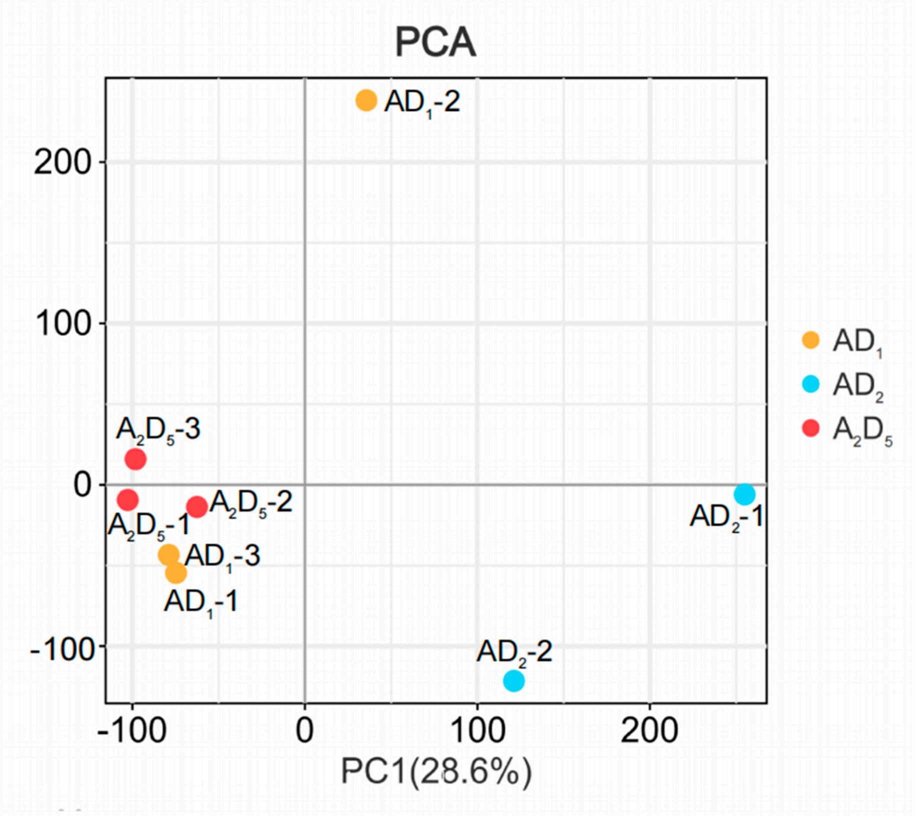

Figure S3. Principal component analysis (PCA) of normalized read counts in TPM.

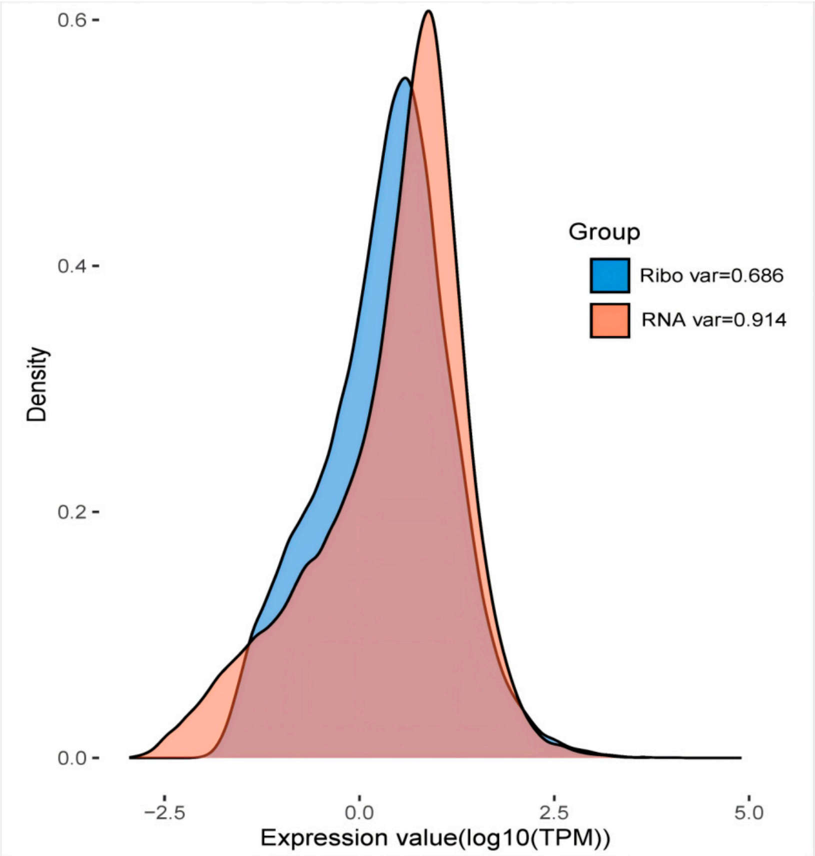

Figure S4. Comparing variances between Ribo-seq and RNA-seq data.

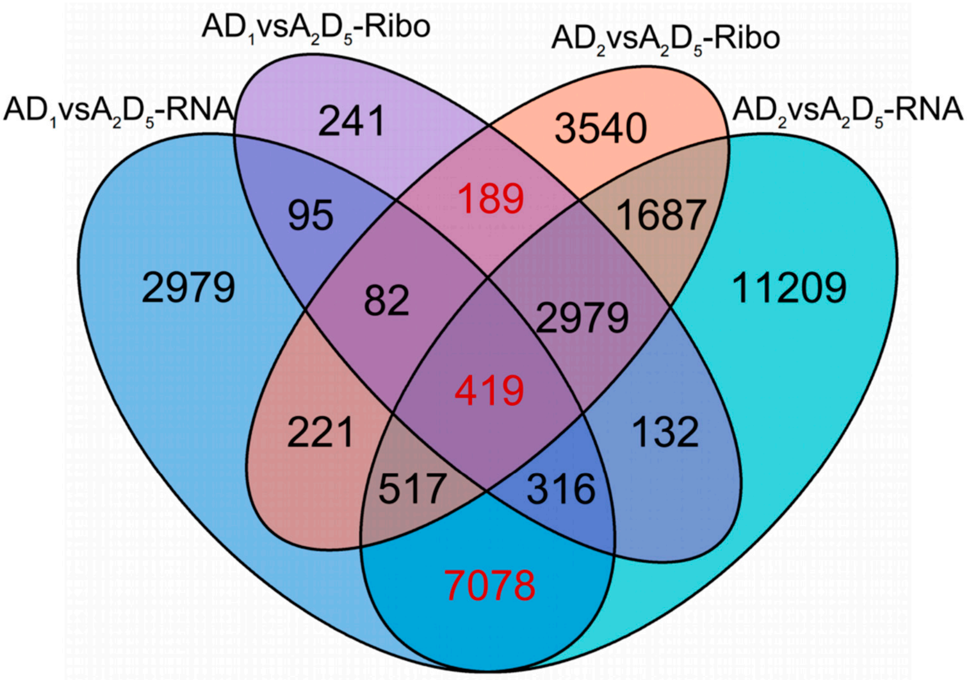

Figure S5. Venn diagram intersection of four DEG lists between diploid and allopolyploid cottons: AD_1 vs A_2D_5 and AD_2 vs A_2D_5 at either transcriptional or translational levels.

Table S1. Primers for the construction of Ribo-seq library

Primers	Sequence
PCR-R-A_2	CAAGCAGAAGACGGCATACGAGAttaggcGTGACTGGAGTTCAGACGTGTGCTCTTCCGATCT
PCR-R-D_5	CAAGCAGAAGACGGCATACGAGAatcacgGTGACTGGAGTTCAGACGTGTGCTCTTCCGATCT
PCR-R-AD_1	CAAGCAGAAGACGGCATACGAGAcgatgtGTGACTGGAGTTCAGACGTGTGCTCTTCCGATCT
RCR-F	AATGATACGGCGACCACCGAGATCTACACGTTCAGAGTTCTACAGTCCGACG

*Note: Lowercase letters - Barcode

Table S2. Ribo-Seq data

Sample	Raw reads	Clean reads	Clean reads rate	The average clean reads rate	Uniq_map reads	Uniq_map rate	The average uniq_map rate
A_2-1	57,010,760	15,388,017	27.0%		5,415,099	35.2%	
A_2-2	60,761,532	17,557,413	28.9%	27.0% (a)	6,115,329	34.8%	35.0% (a)
A_2-3	65,558,930	16,537,991	25.2%		5,769,612	34.9%	
D_5-1	44,021,847	16,547,907	37.6%		5,649,982	34.1%	
D_5-2	40,251,467	10,636,569	26.4%	37.0% (a)	4,209,469	39.6%	34.6% (a)
D_5-3	46,092,333	21,652,671	47.0%		6,508,408	30.1%	
AD_1-1	78,068,856	20,485,294	26.2%		2,299,885	11.2%	
AD_1-2	72,600,499	19,656,057	27.1%	28.4%(a)	3,899,578	19.8%	17.3% (b)
AD_1-3	75,539,268	24,085,635	31.9%		5,001,671	20.8%	

*Note: Different letters in parentheses indicate student t-tests significantly different at the 0.05 level

Table S3. RNA-Seq data

Sample	Raw Reads	Clean Reads	Raw Base(G)	Clean Base(G)	Effective(%)	Error(%)	Q20(%)	Q30(%)	GC(%)	Uniq map reads	Uniq map rate(%)
A_2-1	48,429,142	47,898,218	7.26	7.18	98.9	0.03	97.61	93.5	43.34	41,721,704	87.1
A_2-2	42,824,098	42,120,698	6.42	6.32	98.36	0.03	97.65	93.5	43.33	36,785,728	87.33
A_2-3	39,393,294	38,834,598	5.91	5.83	98.58	0.03	97.66	93.5	42.69	34,157,438	87.96
D_5-1	45,695,760	45,083,272	6.85	6.76	98.66	0.03	97.46	93.09	43.74	38,901,404	86.29
D_5-2	51,937,092	51,112,712	7.79	7.67	98.41	0.02	98.06	94.49	43.35	43,439,174	84.99
D_5-3	45,346,080	44,760,658	6.8	6.71	98.71	0.03	97.62	93.43	42.48	38,026,452	84.96
AD_1-1	57,242,368	56,484,000	8.59	8.47	98.68	0.03	97.88	94.12	43.98	50,461,998	89.34
AD_1-2	40,089,868	39,415,458	6.01	5.91	98.32	0.03	97.4	92.98	42.93	35,039,834	88.9
AD_1-3	57,676,392	56,908,806	8.65	8.54	98.67	0.02	98.06	94.55	43.94	51,192,482	89.96

Table S4. Expressed gene number on Ribo-seq

Ribo	expression（TPM>1）	non-expression	counts>0
AD_1	40,591	12,392	52,983
AD_2	29,491	12,791	42,282
A_2D_5	40,791	14,329	55,120
A_2	23,465	6,292	29,757
D_5	21,922	7,082	29,004

Table S5. Expressed gene number on RNA-seq

RNA	expression（TPM>1）	non-expression	counts>0
AD_1	46,905	15,438	62,343
AD_2	43,291	17,920	61,211
A_2D_5	47,879	17,122	65,001
A_2	25,006	5,860	30,866
D_5	26,356	6,654	33,010
